# Microglia and neuroinflammation: function, heterogeneity, and crosstalk

**DOI:** 10.1038/s41423-026-01438-3

**Published:** 2026-06-04

**Authors:** Shuai Zong, Xiaolin Cui, Shuang Wu, Zhiming Lu

**Affiliations:** 1 Department of Clinical Laboratory, Shandong Provincial Hospital Affiliated to Shandong First Medical University, Jinan, China; 2 Department of Clinical Laboratory, Shandong Provincial Hospital, Cheeloo College of Medicine, Shandong University, Jinan, China

**Keywords:** Microglia, Neuroinflammation, Cellular heterogeneity, Neuroimmune crosstalk, Mechanisms of disease, Inflammation

## Abstract

Microglia, the resident innate immune cells of the central nervous system (CNS), are indispensable for maintaining brain homeostasis, conducting immune surveillance, and responding to injury. Recent single-cell sequencing studies have revealed that activated microglia exhibit a spectrum of activation states that extend well beyond the classical proinflammatory/anti-inflammatory dichotomy, encompassing distinct subpopulations such as disease-associated microglia (DAMs), termed interferon-responsive microglia (IRMs), and lipid-droplet-accumulating microglia (LDAMs). Their remarkable plasticity enables microglia to adopt dual functional roles—either neuroprotective or neurotoxic—depending on the context of neuroinflammatory disease progression. Furthermore, microglia do not act in isolation but serve as central communicators within a dynamic cellular network of the CNS, interacting with neurons, astrocytes, oligodendrocytes, and peripheral immune cells to regulate processes such as synaptic pruning, inflammatory amplification, and myelin integrity and repair. This review provides a comprehensive overview of microglial origin, development, and classification, as well as the dynamic spectrum of microglial cellular states. Furthermore, we discuss the classical and latest mechanisms of microglia-mediated neuroinflammation and focus on the crosstalk between microglia and other cells of the CNS. The hub position of microglia within neuroinflammatory networks, together with their unique cellular characteristics, may unlock a promising frontier for the development of precision therapeutic strategies against neuroinflammatory disorders.

## Introduction

Neuroinflammation within the central nervous system (CNS) serves as a critical defense mechanism that is largely mediated by glial cells, including microglia and astrocytes. This response supports initial neuroprotection by promoting tissue repair and clearing cellular debris. Inflammatory stimuli may persist, including endogenously produced disease-associated molecules [e.g., amyloid-β (Aβ), tau, α-synuclein and APOE genetic variants] and environmental factors (e.g., trauma and infection with non-self-invading pathogens) [1]. A sustained inflammatory response shifts neuroinflammation from beneficial to neurotoxic effects, ultimately suppressing regenerative processes and leading to neurodegenerative disorders [2].

For many decades, microglia were considered inert spectators of CNS physiology on the basis of early observations. With the development of a microglial culture system, microglia were determined to be the primary source of proinflammatory cytokines such as interleukin-1 alpha (IL-1α), interleukin-1 beta (IL-1β), and tumor necrosis factor-alpha (TNF-α) under lipopolysaccharide (LPS) stimulation, which participate in certain pathological states of brain tissue. A pivotal shift in this view occurred in the mid-2000s, when two-photon in vivo imaging revealed that “resting” microglia are highly dynamic cells that continuously survey the brain parenchyma through their motility and rapidly respond to focal injury [3, 4]. At present, microglia are recognized as the prototypical macrophage-like innate immune cells of the CNS that contribute to homeostasis, immune surveillance, and CNS development [5, 6]. In particular, compared with other glial cells, microglia are activated earlier to respond to pathological insults [7]. As revolutionary technologies in biological research, single-cell/nuclear RNA sequencing (scRNA-seq/snRNA-seq) and spatial transcriptomics have provided unmatched precision in analyzing microglial diversity and spatiotemporally regulated profiles [8]. Compared with previously anticipated microglia, activated microglia display more diverse and dynamic functions that enable situation-specific responses [9]. The characterization of well-defined reactive microglial states in adverse contexts of neurodegeneration, including Alzheimer’s disease (AD), Parkinson’s disease (PD), and multiple sclerosis (MS), remains critical [1, 10, 11].

This review summarizes the latest scientific achievements concerning the role of microglia in neuroinflammation. We first systematically outline the evolving understanding of microglial origin, development, and classification. Second, we summarize and discuss recent scientific advances in microglial heterogeneity, with a particular focus on context-dependent mechanisms in neurodegenerative disorders. Third, we focus on the function and mechanism of microglia-mediated neuroinflammation in neurodegenerative disorders. Finally, we highlight the key findings of complex intercellular crosstalk involving microglia during neuroinflammation.

## Origin and development of microglia

The CNS consists of billions of neuroectodermal cells, including neurons, astrocytes, and oligodendrocytes, as well as a highly specialized macrophage population. Under physiological conditions, CNS macrophages can be divided into two major subgroups. Microglia represent the best-characterized and most abundant macrophage population in the brain parenchyma. CNS-associated macrophages (CAMs), also referred to as border-associated macrophages (BAMs), reside at CNS interfaces such as the meninges, perivascular spaces, and choroid plexus [12, 13].

In 1919, Spanish neuroscientist Pío del Río-Hortega first identified a morphologically distinct glial cell population in stained neural tissues. The term “microglia” is derived from the Greek roots *micro* (small) and *glia* (glial cells) and refers to its small cell size and classification within the glial lineage [14]. The embryonic origin of microglia—whether derived from the mesoderm or ectoderm—remained controversial throughout the 20th century. This controversy was resolved by a landmark lineage tracing study [15], which demonstrated that microglia arise from primitive erythroid–myeloid precursors (EMPs), also known as primitive macrophages. In contrast, tissue-resident macrophages in other tissues primarily originate from fetal monocytes differentiated from definitive Myb⁺ EMPs [15, 16].

Mammalian embryonic hematopoiesis represents a sophisticated process driven by multiple temporally overlapping developmental programs [17–20]. In mice, the earliest wave of hematopoiesis, termed primitive hematopoiesis, is initiated in the YS blood islands of the posterior plate mesoderm at embryonic day (E) 7.0 [17, 21]. Primitive EMPs arise from the hemogenic endothelium around E7.5, a process regulated by runt-related transcription factor 1 (RUNX1) [15, 22]. Shortly after the emergence of primitive EMPs, YS-derived EMPs establish the first wave of “definitive” hematopoiesis [19, 23]. At E8.25, these EMPs are phenotypically defined as KIT⁺CD41⁺CD45^low^AA4.1⁺ and can differentiate into erythrocytes and YS macrophages [20, 23, 24]. As early as E9, EMPs migrate to the fetal liver (FL) and participate in FL hematopoiesis, generating F4/80^hi^CD11^blow^ YS-derived macrophages, monocytes, granulocytes, and erythrocytes [24, 25]. Most adult tissue-resident macrophages—including microglia—are derived from Tie2⁺ yolk sac progenitors that give rise to CSF1R⁺ erythro‑myeloid precursors (EMPs), which are distinct from conventional HSCs [20]. At approximately E14.5, abundant FL monocytes infiltrate nearly all peripheral tissues but fail to invade the brain [16]. This exclusion is largely attributed to the blood–brain barrier (BBB), which begins to form at E13.5 and restricts monocyte entry into the CNS [16, 26]. At E10.5, the second wave of “definitive” hematopoiesis is driven by HSCs originating from the hemogenic endothelium of the aorta–gonad–mesonephros (AGM) region, which subsequently migrate to the FL [27, 28]. This evidence supports the notion that HSCs and EMPs arise from distinct hemogenic endothelial cell populations [29]. As early as E9–E9.5, immature HSCs can be detected in the hemogenic endothelium of the para-aortic splanchnopleural (P-Sp) region [19]. FL monocytes derived from HSCs contribute only marginally to the tissue-resident macrophage pool [20]. Under steady-state conditions, YS-derived macrophages are scarcely replaced by HSC-derived cells, with only a minor proportion undergoing such turnover [20].

The direct precursors of microglia are YS macrophages derived from EMPs. At E9, YS c-Kit⁺ EMPs differentiate into immature A1 cells (CD45⁺c-Kitl^ow^CX3CR1⁻) and subsequently mature into A2 microglial progenitors (CD45⁺c-Kit⁻CX3CR1⁺). Interferon regulatory factor 8 (IRF8) and PU.1 (also designated SPI1) are critical for microglial development at both the A1 and A2 stages, either as heterodimerization partners or independently [30]. The loss of PU.1 results in a marked reduction in the number of A1 and A2 progenitor cells, whereas IRF8 deficiency specifically decreases only the population of A2 progenitors in the YS [28, 30]. Starting from E9.5, microglial progenitors migrate from the YS to the rudiment of the brain via intravascular trafficking, a process mediated by the macrophage colony-stimulating factor 1 (CSF1)-CSF1R axis [31]. They then migrate into the leptomeninges and lateral ventricles and subsequently disperse throughout the cerebral cortex. Furthermore, several chemokine-mediated signaling pathways—such as the CX3C chemokine ligand 1/CX3C chemokine receptor 1 (CX3CL1/CX3CR1) [32] and C-X-C motif chemokine ligand 2/C-X-C chemokine receptor type 4 (CXCL2/CXCR4) axes—are also essential for the avascular migration of microglia throughout the developing CNS and their successful colonization.

Notably, scRNA-seq technology allows us to explore the origin and differentiation of human microglia in an unbiased and unsupervised manner while preserving information regarding tissue location and temporal dynamics [33–36]. A study based on a modified single-cell tagging reverse transcription sequencing method (STRT-seq) traced the differentiation trajectories of tissue-resident macrophages, identifying human macrophage differentiation independent of hematopoietic stem cells—similar to that observed in mice—thereby advancing our understanding of the yolk sac origin of human microglia [34]. Although microglia originate from YS-derived macrophage progenitors (YSdMPs), a comprehensive overview of human macrophage developmental dynamics obtained through scRNA-seq revealed that head-enriched macrophage progenitors (HeMPs) may act as a transitional population for microglia [35]. YSdMPs comprise two distinct subpopulations, designated YSdMP_AFP^low^ and YSdMP_AFP^high^. A bifurcating differentiation trajectory was identified starting from YSdMP_AFP^high^: one pathway leads to proangiogenic macrophages (PraMs), characterized by the gradual differentiation of YSdMPs into pre-PraMs and ultimately into mature PraMs; the other pathway leads to microglia, with HeMPs serving as an intermediate stage [35]. Furthermore, emerging evidence indicates that EMPs consist of two primitive EMP (pEMP) subsets—CSF1R⁻ and CSF1R⁺—along with one differentiated EMP (dEMP) subset [33]. Notably, CSF1R⁺ pEMPs are derived from CSF1R⁻ pEMPs. Microglia originate predominantly from pEMPs, whereas dEMPs give rise to tissue-resident macrophages in peripheral organs [33].

## Heterogeneity of microglia: deepening the understanding of neurodegeneration

### Microglial conservation and heterogeneity in an evolutionary framework

New technologies provide a powerful and unbiased approach to describe the transcriptional signature of microglia-specific programs for sensing changes in the brain’s milieu [37]. In mice and humans, microglia express macrophage markers, including CX3CR1, CSF1R, ionized calcium-binding adapter molecule 1 (IBA1), CD11b, crystallizable fragment (Fc) receptors, and surface glycoprotein F4/80 (Adgre1) [38]. Genome-wide transcriptome and epigenome studies revealed that TREM2, TYROBP, CD33, SALL1 and P2RY12 were specifically expressed in mouse microglia but not in other types of resident macrophages or other cells in the brain, suggesting that microglia are highly specialized and have a unique ontogenetic profile. The purinergic receptor P2RY12 and the transmembrane protein TMEM119 were also observed in human microglia. A comparison of the microglial transcriptomes between humans and mice using cells isolated from brain tissues without obvious neuropathological abnormalities revealed a high degree of similarity between the two species. Increased expression in human microglia was associated with the complement system (C1QA, C1QB, C1QC, C2, C3, and VSIG4) and immune pathways (GNLY, CD58, APOBEC3C, CLECL1, CD89, and CARD8) [39, 40]. Nevertheless, markedly low overlap was detected when age-associated transcriptomic changes in microglia from humans and mice were compared, revealing that mouse models of neurodegenerative disorders only partially recapitulate the complex brain environment encountered in patients. Human Alzheimer’s microglia (HAMs), a subset of microglia isolated from the human frontal cortex, are almost entirely distinct from the DAM profile induced in neurodegenerative mouse models [41, 42].

The transcriptomic heterogeneity of microglia must also be examined within an evolutionary framework to determine their broad core functions and to distinguish species-specific functional adaptations [43]. Microglia-like cells with macrophage-like morphology and phagocytic capacity have been documented in the ganglia of invertebrates, suggesting that microglia (or microglia-like cells) represent an ancient component of the innate immune system within the nervous system [44]. In vertebrates, microglia have been identified across diverse species, including teleost fish (e.g., zebrafish), birds, rodents, nonhuman primates, and humans, confirming that microglia emerged as a specialized CNS-resident myeloid cell lineage early in vertebrate evolution [45, 46]. This phylogenetic distribution highlights the developmental conservation of microglia.

Functional and molecular conservation represents another dimension of microglial evolution. Homeostatic microglia have been shown to perform markedly similar physiological functions across species, including immune surveillance of the CNS parenchyma, clearance of apoptotic debris, and synaptic pruning during development and plasticity [45, 47]. At the molecular level, a cross-species transcriptomic analysis spanning more than 450 million years of evolution revealed a conserved core program of orthologous genes expressed in microglia from rodents to humans, including *P2RY12, TMEM119, SALL1, CX3CR1, HEXB*, and *FCRLS* [48].

However, this evolutionary conservation coexists with species-specific divergence. Although core homeostatic functions are maintained, microglial morphology, gene expression levels, and responses to pathological stimuli differ markedly among species, and these differences are thought to reflect evolutionary adaptations shaped by species-specific factors [39, 48, 49]. Cross-species studies have revealed notable differences in complement pathways, phagocytic mechanisms, and signaling cascades enriched with genes associated with susceptibility to brain disorders such as AD and PD [48], highlighting the limitations of rodent models in fully recapitulating human microglial biology in disease contexts.

### Microglial signature during homeostasis

Microglia are pivotal for brain homeostasis throughout life, and their tight regulation prevents deviation and leads to distinct functional effects. Under homeostatic conditions, microglia employ their ramified processes to continuously monitor the microenvironment for potential signals that trigger subsequent functional responses. This surveillance capacity is mediated by a repertoire of surface receptors, collectively termed the microglial “sensome,” which enables the detection of diverse cues, including chemokines, cytokines, purinergic molecules, and amino acids [9, 50].

The molecular identity that underlies these homeostatic functions has been systematically defined through transcriptomic profiling. Microglia isolated from the brain tissues of cognitively healthy humans led to the identification of a gene expression signature specific to homeostatic microglia. Markers of homeostatic microglia include genes that encode microglia-enriched surface receptors, such as *CX3CR1, P2RY12*, and *TMEM119* [51, 52]. Intriguingly, the relationship between homeostatic status and functional responsiveness is not always straightforward. Studies in mouse models have shown that microglia can retain a molecular homeostatic signature while exhibiting a blunted capacity to respond to pathological insults. For instance, microglia isolated from adult TREM2-deficient mice have been shown to maintain a canonical homeostatic state, given that they are less effective as stimuli such as amyloids and fail to develop the typical transcriptional DAM profile in AD [53, 54].

### DAM state and other microglial states in neurodegeneration

Given the cellular heterogeneity exhibited by microglia, scRNA-seq was applied to elucidate the contributions of immune cell subpopulations to the initiation and pathological progression of AD [9]. A novel protective microglial subtype, disease-associated microglia (DAMs), was shown to arise from homeostatic microglia [55]. The DAM signature encompasses the downregulation of homeostatic microglia-specific genes such as *P2RY12, CX3CR1*, and *TMEM119* and the upregulation of distinct marker genes, including *apolipoprotein E (APOE)*, *CTSD*, *LPL*, *TYROBP*, and *TREM2* [40, 56]. The DAM-specific program is triggered by a two-step process: initial activation occurs via a TREM2-independent mechanism that negatively regulates homeostatic microglia checkpoint genes and induces a partial DAM transcriptional signature; the second stage requires TREM2-dependent signaling and involves significant engagement of lipid metabolism, phagocytic pathways, and modulation of the immune response. DAMs predominantly localize around Aβ plaques and may display increased phagocytic activities, such as clearing Aβ, restricting plaque-induced neurotoxicity, and delaying the progression of AD. MERFISH spatial transcriptomics analysis has further characterized the distinct distribution patterns of DAMs relative to plaque density and brain regions, revealing significant enrichment in the corpus callosum and subcortical regions [57]. The DAM transcriptional state appears to be tightly linked to specific pathological conditions and likely represents a conserved core response elicited by diverse challenges [58]. A molecular signature consistent with that of the DAM has been observed in several neurodegenerative disease models, including those of amyotrophic lateral sclerosis (ALS) [55], MS, and AD. This conserved program, also termed the microglial neurodegenerative phenotype (MGnD), is induced by the phagocytosis of apoptotic neurons and has been proposed to exert protective effects in response to neuronal injury in an APOE-dependent manner [59]. Whether individual DAM-associated features, such as the upregulation of immune-reactive genes, reflect adaptive protective mechanisms or, alternatively, maladaptive consequences of chronic phagocytic overload at different disease stages, remains an area of active investigation.

In addition to disease-associated heterogeneity, microglial diversity is profoundly shaped by developmental stage and aging. Early developmental microglia exhibit the greatest functional diversity in terms of cell metabolism, growth, motility, and proliferation. Notably, microglia exhibit substantially increased heterogeneity in early postnatal stages. This heterogeneity gradually diminishes to a more homogeneous state in adulthood [60]. Disruption of this homogeneity occurs when the brain experiences notable changes, including those that take place during aging, pathological processes, and neurodegenerative conditions [61, 62]. With aging, microglia undergo a phenotypic shift to a more neuroprotective profile, in contrast to the peripheral immune system, which may become more predisposed to a neurotoxic state [50, 63].

The diversity of context-dependent microglial states has been shown to involve partially overlapping transcriptional profiles with those of DAMs across different models, diseases, and developmental stages [58]. In the developing brain, several transient populations have been characterized. Axon tract-associated microglia (ATMs), also known as youth-associated microglia (YAM), localize to myelinating regions and upregulate the expression of GPNMB, IGF1, LGALS1/3, LAMP1, and SPP1 [64]. These cells share several markers with DAMs, suggesting that they share activation programs between development and disease. Proliferative-region-associated microglia (PAMs) transiently appear in developing white matter at the onset of myelination, display an amoeboid morphology, and are enriched in metabolic genes involved in oxidative phosphorylation and glycolysis, which is consistent with their role in the phagocytosis of newly formed oligodendrocytes [60]. Activated response microglia (ARMs), identified in both AD models and young wild-type mice, further illustrate that certain activation states are not obligatorily linked to pathology [65].

As the brain ages, microglial heterogeneity re-emerges. White matter-associated microglia (WAMs) upregulate genes involved in phagocytic and lipid metabolic processes [66]. A distinct age-related subset, lipid-droplet-accumulating microglia (LDAM), exhibits impaired phagocytic capacity, elevated levels of reactive oxygen species, and increased production of proinflammatory cytokines [67]. Aging microglia also give rise to small, localized inflammatory populations, termed interferon-responsive microglia (IRMs), which are associated with age-related brain inflammation. Notably, IRM enhances neuronal engulfment, which restricts the accumulation of DNA-damaged neurons from postnatal day 5, revealing that an antiviral-like signaling program can be coopted for homeostatic functions during development [68]. Disease-specific microglial states have also been described in chronic neurodegenerative and neuroinflammatory disorders. In MS, microglia inflamed in MS (MIMS) display a transcriptional program specialized for myelin phagocytosis and clearance, along with antigen presentation and the propagation of inflammatory damage [69, 70]. Distinct microglial signatures have likewise been reported in ALS [71] and PD [72, 73].

The identification of the heterogeneity of microglia in mouse models and the human brain deepens our insight into microglial biology. These cells are not permanently confined to any single functional state. The dynamic spectrum of microglial cellular states facilitates their ability to adapt to and face challenges. Several studies have indicated that the transition of microglia toward the DAM initiates early in the disease process and intensifies with disease progression. Direct comparisons of these DAMs indicate a robust correlation among their subpopulation signatures across diverse contexts. Interestingly, microglia still display highly context-specific gene expression changes to fulfill specialized functions beyond the core DAM signature.

### Microglial cross-tissue heterogeneity: from CNS residency to peripheral distribution

Studies in mice have provided key insights into the embryonic origins and differentiation characteristics of a variety of tissue-resident macrophages, including microglia in the CNS, hepatic Kupffer cells, epidermal Langerhans cells, bone osteoclasts, and intestinal macrophages [20, 74, 75]. Microglia are an integral component of the CNS and have long been regarded as being absent from the peripheral nervous system (PNS) [76, 77]. Single-cell genomics has revealed cellular diversity with unprecedented precision, challenging the long-standing notion that certain cell types exhibit strict tissue specificity [35, 76–78]. The discovery that immune cell populations outside the brain and spinal cord share the transcriptomic signatures and ontogeny of microglia has sparked a profound conceptual shift in this field.

Comprehensive mapping of human macrophage heterogeneity using scRNA-seq has revealed the presence of microglia-like cells expressing core microglial marker genes (*P2RY12, TMEM119, SALL1*, and *C3*) outside the CNS [35]. These cells, which display transcriptomic profiles and morphological features analogous to those of CNS microglia, are distributed in the fetal epidermis, testes, and heart. Both microglia and microglia-like cells in these tissues exhibit diverse morphologies (spherical, amoeboid, and branched), with cells in the CNS and epidermis showing a more prominent branched/mature phenotype than those in the heart and testes do. Among these tissues, microglia-like cells are most abundant in the epidermis, emerging as early as the Carnegie stage 12 (CS12), when they interact with neural crest cells to regulate their differentiation into the melanocyte lineage. In the heart, microglia-like cells first appear at the CS13 stage and subsequently accumulate gradually in the aorta. In the testes, these cells initially emerge at the CS14 stage around the mesonephric tubules—a structure that later develops into the efferent ducts. However, their specific functions in the testes and heart remain to be fully elucidated [35]. Another study demonstrated that macrophages and endothelial cells can colocalize with neurons in the “early and late neurovascular microenvironment,” thereby contributing to early human skin morphogenesis, including scarless skin repair, fibroblast homeostasis, and neurovascular development [79]. These functions are partially attributed to YS-derived TREM2+ macrophages, whose expression profiles (P2RY12, CX3CR1, and OLFML3) are similar to those of microglial-like macrophages in other developing organs (e.g., the brain, prenatal skin, and gonads) [80]. TREM2+ microglial-like (TML) macrophages in prenatal skin are highly related to microglia in the embryonic brain and may endow early fetal skin with the unique property of scar-free healing. Notably, TML macrophages colocalize with Schwann cells in prenatal skin (within the “early neurovascular microenvironment”) and express genes associated with cell migration and neurodevelopment, participating in synapse formation and axon guidance. These findings suggest that macrophages in early-pregnancy skin may play a supportive role in the establishment of the PNS within the skin [79, 81]. TREM2+ macrophages with microglial-like characteristics (expressing TREM2, P2RY12, and SALL1) have also been identified in the fetal testis [34, 78]. In contrast to tissue-repair macrophages, which are ubiquitous across all developing tissues, TREM2+ fetal testicular macrophages represent a rare population, accounting for approximately 5% of the total macrophage pool, and are primarily localized within the testicular cords. It is hypothesized that these macrophages may communicate with supporting cells and germ cells through interactions between TREM2 and apolipoproteins (CLU, APOA1, and APOE), thereby contributing to the maintenance of the immunoregulatory microenvironment previously described in the testis [78, 82].

A recent study revealed a population of tissue-resident macrophages within the PNS that share common developmental trajectories, molecular signatures, and epigenetic features with human microglia; these cells are termed PNS microglia [83]. The presence of PNS microglia is positively correlated with body size and the size of peripheral neuronal cell bodies. Specifically, PNS microglia are present in humans, macaques, and pigs but have not been detected in animals with smaller body sizes and smaller neuronal cell bodies, such as mice and rats. These findings explain the absence of PNS microglia in commonly used rodent models and highlight the significance of broader cross-species analysis in advancing this field. During development, PNS microglia, similar to CNS microglia, differentiate from YS-derived macrophage progenitor cells via an MRC1 + P2RY12+ precursor population. Previous studies in rodents have demonstrated that satellite glial cells (SGCs) envelop neuronal somata to form sheath-like structures [84]. In contrast to the current understanding, the results of this recent study suggest that in the peripheral ganglia of humans, macaques, and pigs, neuronal cell bodies are first enveloped by PNS microglia, which are subsequently surrounded by SGCs, ultimately forming a ternary cell structure consisting of “neuron–PNS microglia–SGC”. Notably, these PNS microglia play key regulatory roles in neuronal soma enlargement and axon growth during PNS development. Similarly, comprehensive profiling of the cross-tissue transcriptional heterogeneity of macrophages in healthy pregnant sows revealed that microglia represent the dominant cell type in the CNS [85]. Distinct microglial subtypes have also been identified in extra-CNS tissues. These findings align with those of human studies in which peripheral microglia are detected in the epididymis, an IFIT1⁺ microglial subtype is present in peripheral blood, and additional microglial subtypes are heterogeneously distributed throughout the male reproductive and urinary systems.

Collectively, single-cell genomic approaches have enabled unbiased cell classification on the basis of molecular signatures and developmental origins, reshaping our knowledge of cell-type distribution across tissues and introducing considerable ambiguity in conventional cell nomenclature. A pressing challenge remains in establishing a unified classification framework for cells that share the same lineage, molecular phenotypes, and developmental origins yet are broadly distributed across diverse tissues. To address this issue, several researchers have proposed the term “microglia lineage” to unify classical CNS-resident microglia and their peripheral counterparts [77]. This nomenclature standard effectively resolves current naming discrepancies, conforms to modern cell taxonomy criteria, and highlights the evolutionary continuity and ontological homology of the microglial lineage [77].

## Microglia mediate neuroinflammation in neurodegenerative disorders

**Fig. 1:**
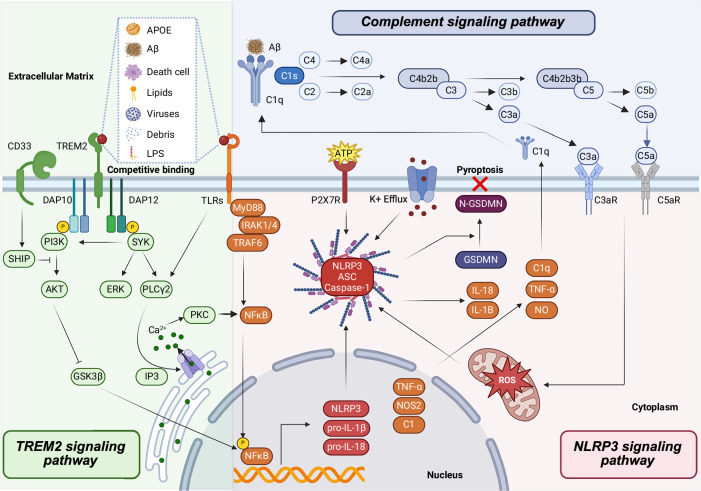
Neuroinflammatory Landscape of Microglia: Integrated Signaling of TREM2, NLRP3, and the Complement System. Microglia utilize a diverse repertoire of surface receptors to sense and respond to pathological cues within the central nervous system. (Left) Aβ oligomers, APOE, and other damage-associated molecular patterns (DAMPs) interact with TREM2, triggering a signaling cascade involving DAP12 and SYK. This pathway activates PLCγ2 and ERK, modulating microglial survival and phagocytic activity. In contrast, CD33 functions as an inhibitory checkpoint, recruiting SHIP to antagonize PI3K/AKT signaling. (Center) Toll-like receptors (TLRs) recognize Aβ and lipopolysaccharide (LPS), transducing signals through the MyD88/TRAF6 axis to induce nuclear translocation of NF-κB. This transcriptional priming upregulates the expression of proinflammatory precursors, including NLRP3, pro-IL-1β, and pro-IL-18. Extracellular ATP activates the P2X7R purinergic receptor, which is coupled with K⁺ efflux to trigger the assembly of the NLRP3 inflammasome (comprising NLRP3, ASC, and caspase-1). Activated caspase-1 processes pro-cytokines into their mature forms (IL-1β and IL-18) and cleaves gasdermin D (GSDMD) to form membrane pores, ultimately leading to pyroptosis. (Right) The complement cascade is initiated by Aβ binding to C1q, leading to the sequential activation of C4, C3, and C5. Anaphylatoxins C3a and C5a bind to their cognate receptors C3aR and C5aR, respectively, further amplifying the inflammatory response and promoting mitochondrial reactive oxygen species (ROS) production. The figure was created with BioRender.com

Neuroinflammation is involved in a broad spectrum of medical disorders, including autoimmune neurological disorders (e.g., MS), acute CNS injuries (e.g., strokes), and neurodegenerative diseases (e.g., AD and PD) [86–88]. As disease progresses, microglia play dual roles as neuroprotective and neurotoxic effectors, with a shift from mediating immune protection to regulating neuroinflammation [89]. The state and activity of microglia serve as criteria for determining the presence of neuroinflammation. In this section, we highlight the similarities in microglial activation characteristics and heterogeneity due to disease-specific factors across neurodegenerative disorders (Fig. 1).

### TREM2-related signaling transmission network

Unlike other types of neuroinflammatory diseases, the core pathology of neurodegenerative diseases involves the deposition of pathological insoluble protein aggregates. These persistently insoluble aggregates are difficult to completely clear, ultimately inducing chronic neuroinflammation. Notably, the ongoing activation of microglia through the phagocytosis of pathological proteins profoundly influences disease progression in patients with neurodegenerative diseases. The typical pathological features of AD include Aβ deposition, neurofibrillary tangles, synaptic dysfunction, and neuroinflammation [90]. Multiple genome-wide association studies (GWASs) have shown that more than 50% of gene variants at AD risk loci are involved in microglial function and are essential for the innate immune and inflammatory responses in AD pathogenesis [91–94]. One of these risk genes, *triggering receptor expressed on myeloid cells 2* (*TREM2*), encodes an immunomodulatory transmembrane receptor primarily expressed in microglia that serves as a core signature of the DAM-specific activation program. Evidence based on TREM2-related studies has focused mainly on phagocytosis and the regulation of anti-inflammatory effects. Notably, many distinct risk genes, such as *APOE, CD33, PLCG2, INPP5D*, and *MS4A6A/MS4A6E*, share potential nodes that cross with TREM2 signaling [95].

Since *TREM2* lacks an intrinsic signaling domain, its activation depends on binding to the adaptor protein DAP12 (also known as TYROBP) by phosphorylating the cytoplasmic immunoreceptor tyrosine-based activation motif (ITAM). This interaction leads to the recruitment of spleen tyrosine kinase (SYK) to regulate downstream signaling cascades, such as the PI3K/AKT and NF-κB signaling pathways. Recently, the phospholipase-encoding gene *PLCG2*, which encodes the protein PLCγ2, was shown to act downstream of *TREM2*, modulating the phagocytosis of Aβ and neuronal debris as well as lipid metabolism [96, 97]. Microglia deficient in SYK may disrupt the transmission of intracellular signals mediated by the TREM2-DAP12 complex [98]. TREM2 is also associated with DP10-dependent signaling mechanisms, leading to the transmission of a variety of intracellular signals [98, 99]. The AD potential risk allele *CD33* encodes a protein containing an immunoreceptor tyrosine-based inhibition motif (ITIM) domain, which is an inhibitor of ITAM, therefore potentially suppressing TREM2 signaling. TREM2 acts downstream of CD33 to regulate cognition, amyloid pathology, and microgliosis via the IL-1b/IL-1RN axis [100]. SH2 domain-containing inositol 5-phosphatase 1 (SHIP1), encoded by another AD risk gene, *INPP5D*, exerts an inhibitory effect on TREM2 signaling by competitively binding to DAP12. A considerable number of studies suggest that SHIP1 represses PI3K signaling and induces the activation of the NLRP3 inflammasome [92, 101]. The potential of a variety of TREM2 ligands, including Aβ [102], bacteria, lipoteichoic acids, anionic lipids (e.g., phosphatidyl serine), and apolipoproteins (e.g., APOE) [103], has been elucidated. Interestingly, compared with Aβ monomers, the affinity of TREM2 for Aβ oligomers is greater. APOE, which has been clearly verified to have the strongest effect on AD pathogenicity (reviewed by Raulin et al.) [92, 104], directly binds to TREM2. A novel study utilized spatial transcriptomics to demonstrate that the upregulation of TREM2 and APOE in microglia contributes to immune-mediated Aβ clearance [105]. MS4A6A is a membrane protein with four transmembrane domains that is localized to endosomal and lysosomal membranes. MS4A6A deficiency has been shown to significantly attenuate the ability of microglia to envelope and phagocytose amyloid plaques and mediate microglial pyroptosis [106]. New evidence implies that MS4A4A and MS4A6A are cooperatively involved in the negative regulation of TREM2 posttranscription and microglial function, blocking the activation of DAP12 [107].

The TREM2 p.R47H variant not only represents a robust risk factor for AD but also confers an increased risk for ALS, PD, and frontotemporal dementia (FTD) [108]. In AD model mice, the heterozygous Trem2 R47H allele results in impaired clearance responses to amyloid deposition, accompanied by attenuated microglial proliferation surrounding plaques [109]. Increased TREM2 expression moderately reduces soluble phosphorylated tau levels and slightly mitigates neurodegeneration [110]. TREM2 deficiency increased the spread of tau in plaque-associated dystrophic neurons, exacerbating Aβ deposition [111]. The TREM2 p.R47H variant represents a robust risk factor for sporadic ALS patients [89].

In contrast to those in AD, the core pathological protein in PD is α‑synuclein, and the affected sites are mainly dopamine‑producing neurons in the nigrostriatal pathway [112]. Microglia can phagocytose α-syn fibrils and convert them into more toxic variants, while TREM2-dependent phagocytosis of α-syn fibrils facilitates the spread of α-syn in the brain, thereby increasing its pathogenicity in PD [113]. Triggering TREM2 expression attenuated neuroinflammation and had strong neuroprotective effects through PI3K/Akt signaling after intracerebral hemorrhage in mice with ischemic stroke [114].

The function of TREM2 is primarily anti-inflammatory and neuroprotective in neurodegenerative disorders; however, it may also exert a proinflammatory effect at distinct disease stages. For instance, the interaction between TREM2 and Aβ can increase the phagocytosis and clearance of Aβ, thereby ameliorating cognitive impairment in AD. Furthermore, the binding of TREM2 to Aβ can trigger PLCγ2-mediated inflammatory responses, which in turn exacerbate Aβ deposition [96]. A similar dichotomy is observed in TREM2-mediated responses to α-syn in PD. Thus, whether TREM2 acts as a proinflammatory or anti-inflammatory regulator is context-dependent and varies with disease stage.

### Inflammasome-mediated signaling cascade

**The** NLRP3 inflammasome, which is expressed primarily by microglia within the CNS, is currently the most well-characterized inflammasome [92]. A novel study confirmed that the deposition of Aβ initiates the activation of the NLRP3 inflammasome and is involved in the development of a DAM-specific program in the pathogenesis of AD [115]. Exogenous ligands (e.g., LPS) or endogenous ligands (e.g., HMGB1 released by damaged neurons, TNF‑α or IL‑1β derived from microglia, or astrocytes themselves) act on corresponding receptors on the microglial surface (e.g., TLR4, TNFR, and IL‑1 R), activating the downstream NF‑κB signaling pathway [116–118]. The core role of this pathway is to induce the transcriptional upregulation of key proteins such as NLRP3 and pro‑IL‑1β, providing the necessary molecular basis for inflammasome assembly [119]. A diverse group of factors, including insoluble protein aggregates, lysosomal disruption caused by the phagocytosis of pathogen‑associated components, the activation of the ATP/P2X7R pathway due to neuronal damage [120, 121], and K^+^ efflux mediated by elevated ROS from cellular stress, collectively disrupt intracellular ion homeostasis, triggering the oligomerization of NLRP3 proteins to form an active inflammasome platform [122]. The assembled inflammasome subsequently recruits and activates caspase‑1. On the one hand, caspase‑1 processes pro‑IL‑1β and pro‑IL‑18 into mature, active IL‑1β and IL‑18 for secretion [123]. On the other hand, caspase‑1 cleaves the GSDMD protein, whose N‑terminal domain forms pores in the cell membrane, inducing pyroptosis. Activation of the NLRP3-GSDMD pathway is necessary for BBB disruption induced by peripheral inflammation through the upregulation of CXCL chemokines and matrix metalloproteinases independent of IL-1β [124]. Robust activation of the NLRP3 inflammasome and the subsequent induction of pyroptosis were moderately correlated with reduced neuronal density in the CA1 region of patients with AD [125]. Notably, after pyroptotic rupture, the release of inflammatory components, including IL‑1β, IL‑18, HMGB1, and ATP, forms a positive feedback loop, significantly amplifying the neuroinflammatory cascade [126] and exacerbating neuroinflammatory diseases. Enhanced activation of NLRP3 has been detected in patients with PD, driving the death of dopaminergic neurons [127, 128]. Chen et al. recently reported that NLRP3 deficiency significantly mitigated α-syn-induced neuronal senescence by inhibiting the activity of the SATB1/DNA damage/p21 axis [129]. For an in-depth review of NLRP3, see Xu et al. [130] and Paik et al. [131].

### Complement system and cooperative crosstalk with other inflammatory mediators

In autoimmune neuroinflammatory conditions such as MS, microglia exhibit an autonomous immune activation state, while marked complement system activation leads to excessive phagocytosis of myelin and synapses [132]. Mechanistically, C1q overexpression on target cells serves as a recruitment signal for phagocytosis by microglia [70, 133]. However, recent studies have revealed that microglia can autonomously secrete C1q [134]. Multiple studies have shown that complement C1q is often cosecreted by activated microglia alongside IL-1β and TNF-α [135, 136]. It has been demonstrated that the combined action of these three molecules on astrocytes drives them toward a neurotoxic reactive state characterized by a loss of homeostatic functions and a gain of a proinflammatory secretory profile [137]. This conclusion has been corroborated by multiple subsequent studies [138, 139]. Consequently, the classical complement cascade initiated by C1q plays a central role in the global coordination of neuroinflammation within the CNS. C1q forms complexes with Aβ [140] and phosphorylated tau [141], triggering the classical complement activation pathway. Conformational changes in C1q induced by binding to pathological proteins activate C1R. C1R subsequently cleaves and activates C1s, which further cleaves component C4 into C4a and C4b. C4b recruits C2, and C1s cleaves C2 into C2a and C2b. At this point, C4b and C2b form C3 convertase (C4b2b), which possesses C3 cleavage activity. Its cleavage products are C3a and C3b, while C3b covalently binds to C4b2b to form C5 convertase (C4b2b3b) with C5 cleavage activity, cleaving C5 into C5a and C5b [142]. The receptor for C3aR, a product of this classical complement activation pathway, is expressed primarily on microglia and belongs to the A class of the G protein-coupled receptor (GPCR) family [143, 144]. The interaction between these two molecules has been shown to cause mitochondrial damage and ROS release in microglia, thereby activating the NLRP3 signaling pathway and leading to cytokine release and pyroptosis in microglia [145]. This phenomenon has been confirmed in multiple diseases, including depression, AD, and ischemic stroke [146, 147]. However, the intracellular signaling pathway of GPCRs mediated by C3aR that leads to this phenotype remains unclear. Elucidating this mechanism may provide insights for the development of anti-inflammatory drugs.

Elevated levels of C5a, another key product of the classical complement activation pathway, have been observed in acute brain injury, ALS, and AD [148–150]. Like C3a, its receptor C5aR is also localized to microglia and exerts comparable effects on microglial activation. A C5aR1+ microglial subpopulation, which drives neurotoxic astrocyte polarization and exacerbates neuroinflammation, was identified in an acute brain injury model [148]. In summary, we propose a novel neuroinflammatory amplification circuit in which inflamed microglia release IL-1β, TNF-α, and C1q to mediate proinflammatory activation of astrocytes, with C1q additionally activating the classical complement pathway to produce C3a and C5a, which act on the microglial receptors C3aR and C5aR; through GPCR-mediated intracellular signaling, this pathway induces mitochondrial ROS release, subsequent NLRP3 pathway activation, and microglial pyroptosis. This process leads to the further release of inflammatory mediators such as IL-1β and TNF-α, along with C1q, ultimately exacerbating neuroinflammation and disease progression. For an in-depth review of cytokines, see Becher et al. [151].

## Interactions of microglia

**Fig. 2:**
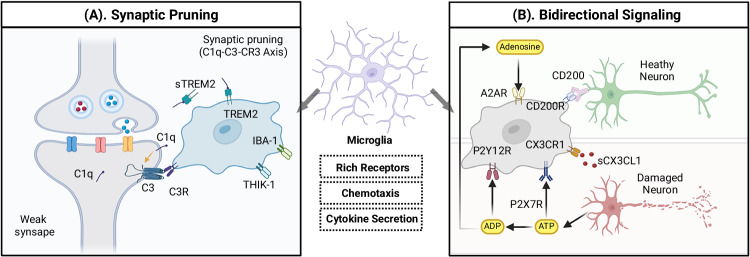
Mechanistic insights into microglial functions in synaptic pruning and neuromicroglial bidirectional signaling. **A** Synaptic pruning via the C1q-C3-CR3 axis. Microglia selectively eliminate weak or redundant synapses through a complement-dependent pathway. Complement component 1q (C1q) tags weak synapses, which are then recognized by complement receptor 3 (CR3) on the microglial membrane. Key microglial markers and receptors, including TREM2 (and its soluble form sTREM2), IBA-1, and THIK-1, are involved in this phagocytic process. **B** Bidirectional signaling between microglia and neurons. Microglia maintain brain homeostasis through complex interactions with healthy and damaged neurons. Top panel: In the healthy state, neurons provide inhibitory “off” signals, such as the CD200–CD200R interaction and adenosine-A2AR signaling, to maintain microglia in a homeostatic state. Bottom panel: Upon neuronal damage, “on” signals (e.g., ATP/ADP and CX3CL1) are released. These molecules bind to purinergic receptors (P2Y12R and P2X7R) and CX3CR1 on microglia, triggering chemotaxis and inflammatory responses. Central panels highlight the fundamental properties of microglia, including rich receptor expression, chemotactic motility, and cytokine secretion. The figure was created with BioRender.com

As resident monitors of homeostasis in the CNS, microglia are equipped with a diverse array of membrane receptors that enable them to respond to various signals. Their chemotactic migration facilitates direct membrane-to-membrane contact with other cell types, while their robust secretion of cytokines grants them potent intercellular communication abilities [152]. In the context of neuroinflammation, these abilities of activated microglia become amplified, leading to their extensive involvement in communication among diverse cell types within the CNS (Fig. 2).

### Microglia–neuron cross-talk

#### Synaptic pruning and microglial complement system activation

A typical neuron comprises a cell body, dendrites, and an axon. The terminal end of an axon forms a synapse with the dendrite or cell body of another neuron. These interconnected synapses collectively form neural circuits, which execute the ultimate function of neural projection [153]. Thus, synapses function as the fundamental structural units for information transmission and processing in the nervous system.

The unique patterns of synaptic number and maturation during neurogenesis were first elucidated in the 1980s. Specifically, neonatal synaptic density is comparable to that of adults, but the synaptic morphology remains immature. Although the timing of synaptic quantity and morphological changes varies across brain regions, the overall synaptic density increases to approximately 1.5 times the adult level by approximately age 2. Subsequently, between the ages of 2 and 16, both the synaptic density and the number of neurons gradually decrease to adult levels [154, 155], accompanied by the progressive maturation of synaptic structures. This peak-and-trough pattern of quantitative change necessarily involves synaptic pruning, and the maturation of neural circuits likely requires external functional support. However, although video-enhanced microscopy revealed prolonged contact between presynaptic terminals and glial cells—suggesting potential glial involvement in synaptic remodeling [156]—the “use it or lose it” principle governing the neurons themselves remained the default synaptic pruning mechanism [157]. In 2009, Wake et al. used in vivo two-photon imaging to demonstrate that microglial processes make intermittent contacts with synapses at approximately 5-min intervals in the healthy brain, with these contacts becoming markedly prolonged after ischemia. Although the rate of synapse disappearance did not differ between contacted and noncontacted synapses, this study provided the first direct in vivo evidence that microglia actively survey synaptic elements [158]. In 2011, Paolicelli et al. demonstrated causality using mice lacking the chemokine receptor CX3CR1 (CX3CR1-KO). These mice exhibited a transient reduction in microglial numbers and a consequent impairment in synaptic pruning, establishing that microglia-mediated synaptic pruning is essential for normal brain development [159]. In the following year, Schafer et al. provided a definitive answer at the molecular level, identifying the C1q-C3-CR3 complement system in microglia as critical for synaptic recognition and clearance. Specifically, C1q marks synapses for pruning, activating the complement cascade to form C3 tags. Microglia recognize and phagocytose these synapses through their surface receptor CR3. Blocking CR3 leads to defective synaptic pruning, resulting in visual dysfunction [160]. This mechanism has been further supported and confirmed by subsequent studies, which have partially elucidated the pathogenesis of multiple diseases involving both neuroinflammation and cognitive decline. In frontotemporal dementia patients, deficiency of the granulin (GRN) gene leads to excessive complement system activation. This results in excessive pruning of inhibitory synapses and hyperexcitability in thalamocortical circuits, ultimately producing compulsive-like phenotypes [161]. In the context of the cognitive impairment caused by neuroinvasive West Nile virus infection, C1QA expression is upregulated and is localized to infected neurons and presynaptic terminals, which are subsequently phagocytosed by microglia. However, synaptic terminals are preserved when complement C3 or C3a receptors are deficient or when IL-34 is knocked out [162]. Furthermore, schizophrenia-associated mutations in the C4 locus and overexpression of the schizophrenia susceptibility factor C4A have been shown to cause complement deposition and excessive synaptic pruning in neurons [163, 164]. Conditional knockout of C1q in microglia prevents neuroinflammation and cognitive impairment induced by radiation therapy for CNS tumors [165] as well as neuropathic pain [166]. Similarly, C5aR1 knockout rescues excessive presynaptic loss in AD mouse models [167]. Additionally, multiple microglial inflammatory factors—including upstream regulators of the complement system, such as SHIP1, IDO1, and STING—have been individually demonstrated to participate in synaptic pruning via the complement system. This finding further reinforces the central role of the complement system in microglial synaptic pruning [168–170].

The precise cellular mechanisms through which microglia ultimately eliminate synapses remain unclear. Microglia are known to phagocytose synaptic material, yet whether they actively “prune” (i.e., select and engulf) intact, functional synapses or primarily “scavenge” synaptic debris already slated for removal by neuronal mechanisms remains controversial [160, 171, 172]. The recent “culling versus scavenging” model proposed by Pereira-Iglesias et al. formally distinguishes these two processes: culling involves the active selection, cleavage, and removal of unwanted synapses, whereas scavenging is a neuron-driven process in which synapses are first shed by neurons and then secondarily cleared by microglia [173]. Nevertheless, changes in microglial phagocytic function inevitably affect this process. For instance, the THIK-1 K⁺ channel governs microglial phagocytic function, and impaired expression of this channel similarly leads to reduced synaptic pruning capacity [174]. Similarly, the expression of IBA1, a microglial marker protein, weakens phagocytic function upon knockout, accompanied by reduced synaptic pruning capacity [175]. Furthermore, increased HMGB1 secretion during neuroinflammation enhances microglial phagocytosis, and suppressing its expression reverses abnormal excitatory synaptic pruning in the hippocampus [176]. Knockout of the schizophrenia-associated gene *CYFIP1* impairs microglial motility, thereby disrupting synaptic pruning processes [177].

Particular attention should be given to TREM2, a key marker of the DAM, which has been demonstrated to participate in multiple functions, including activation control, chemotaxis, and phagocytosis [95, 178]. In 2018, Filipello et al. reported that TREM2 deficiency impaired synaptic clearance [179]. Mechanistically, the authors attributed the role of TREM2 in synaptic pruning to its phagocytic function. However, Zhong et al. recently revealed a physical interaction between TREM2 and C1q. Under physiological conditions, secreted TREM2 carries C1q for controlled synaptic pruning. However, when TREM2 expression decreases, C1q loses its adhesive function and becomes dysregulated, leading to excessive pruning [180]. These findings therefore suggest that future studies on synaptic pruning should include validation of target molecules in relation to the complement system to ensure a comprehensive characterization of their molecular functions.

#### Determination of the glial state via neuronal cytokines

Healthy neurons are thought to maintain microglia in a quiescent state through constitutive signaling, thereby preventing excessive neuroinflammation under physiological conditions. Conversely, neuronal stress or injury can trigger the release of chemotactic factors that recruit microglia and promote their interaction with affected neurons. A series of studies have investigated how neuronal cytokines and compounds regulate microglial activity through contact-dependent or contact-independent mechanisms.

The neuronal chemokine CX3CL1 and its microglial receptor CX3CR1 constitute the core signaling axis of this process. CX3CL1 is a transmembrane glycoprotein expressed on neurons that also exists in a soluble secreted form. This dual nature enables it to function as both a chemokine and an adhesion molecule: it can be secreted to chemoattract microglia over long distances or exist in a membrane-bound form to directly mediate stable adhesion between neurons and microglia. Its sole receptor, CX3CR1, is expressed on the microglial cell membrane. CX3CR1 knockout mice exhibit spontaneous microglia-mediated neurotoxicity [181]. When neuronal CX3CL1 expression is upregulated, or its secretion is increased, microglia receive signals via CX3CR1 and undergo chemotactic migration toward neurons, accompanied by elevated intracellular calcium levels, to initiate microglial activation [182].

Additionally, neurons transmit inhibitory signals through other membrane proteins as a balancing mechanism. The neuronal membrane glycoprotein CD200 and its glial receptor CD200R constitute a critical immunosuppressive checkpoint pathway. Deficiency in CD200 expression significantly enhances microglial reactivity, ultimately exacerbating the pathological progression of autoimmune encephalomyelitis [183], whereas knockdown of CD200R expression reduces microglial responsiveness [184, 185].

In recent years, additional cytokines have been identified as neuron-derived molecules that regulate microglia, including the proinflammatory signals IL-1α and IL-6, the anti-inflammatory signal IL-13, and chemotactic signals via the CXCL/CXCR pathway [186, 187]. Collectively, these studies reveal that neurons dynamically regulate microglial activation states through a finely tuned, reversible cytokine signaling system (e.g., CX3CL1-mediated activation and CD200-mediated inhibition), thereby balancing homeostasis maintenance with demand-driven responses.

#### Rapid glial responses rely on neuron-derived purinergic and metabolite-mediated signaling

Protein-mediated signaling requires sequential processes, including gene transcription, mRNA translation, and protein modification, transport, and release. This lengthy response time fails to meet the immediate needs of neurons during acute injury. In contrast, neuron-derived compound-mediated glial activation offers a speed advantage. The receptor‒axon axis of the ATP/adenosine–purinergic signaling pathway is key for rapid microglial responses.

During neuropathic injuries such as ischemic stroke, ATP is massively released from damaged neurons into the extracellular space. This extracellular ATP binds to P2 receptors on microglia, triggering their activation and mediating neuroinflammation [188]. Multiple P2 receptors that mediate microglial activation and migration have been identified. Among these, activation of the ligand-gated ion channel receptor P2X7R mediates NLRP3 inflammasome assembly and the mature release of the proinflammatory cytokines IL-1β and IL-18 [189, 190]. In contrast, the knockout of another ATP-responsive molecule, P2X4R, enhances the phagocytic activity of microglia [191, 192].

Following the rapid response mediated by ion-channel-type purinergic receptors (P2Xs), the relatively slower-acting G protein-coupled purinergic receptor P2Y12R is activated by adenosine diphosphate (ADP), a hydrolysis product of ATP. Through its coupled Gi protein, P2Y12R mediates microglial migration, ultimately supporting or processing damaged neurons [193–195].

The duration and extent of microglial activation require stringent negative feedback control, with the adenosine/P1 axis serving as the core regulatory pathway—a process that has now been well elucidated. ATP released from damaged neurons is first hydrolyzed by the extracellular enzyme CD39 into ADP, which is subsequently converted to adenosine monophosphate (AMP). Under the action of CD73, AMP is further hydrolyzed into adenosine [196], which acts on A2ARs—a G protein-coupled receptor that specifically binds adenosine and is highly expressed on microglia [197, 198]. A2AR subsequently activates its coupled G protein, which in turn stimulates adenylate cyclase. This leads to a significant increase in intracellular cyclic adenosine monophosphate (cAMP) levels, ultimately resulting in P2Y12 downregulation. This process limits microglial activation and migration [199–201].

In recent years, several non-ATP-related neuronally derived compounds have also been shown to participate in microglial activation and inhibition. For instance, NADPH acts as an endogenous P2X7 modulator by competitively binding to ATP, thereby blocking NLRP3-mediated microglial activation [202], whereas UDP can mediate microglial migration by activating P2Y6R [203]. These nonclassical activation and inhibition signals complement each other, collectively ensuring the temporal and spatial control of microglial activation following acute neuronal injury.

### Microglia‒astrocyte cross-talk

#### Synaptic homeostasis maintenance via immunoregulatory signaling

Astrocytes form synapses with neurons through physical contact to receive signals and provide neuronal support [204], whereas microglia, as resident immune cells, are responsible for phagocytosing and clearing redundant or damaged synapses [205]. The previously held view of their independent functions has shifted because of recent studies, with current research supporting close functional coupling between astrocytes and microglia. Specifically, resident astrocytes participate in neuronal synaptic pruning through interactions with microglia.

First, astrocytes initiate complement-dependent recruitment signals. Specifically, BST2 in astrocytes accumulates at injury boundaries and promotes C3 expression through PKCβII phosphorylation, thereby recruiting microglia for synaptic pruning [206]. Second, the dynamic adjustment of physical space represents another mechanism through which astrocytes are involved in synaptic pruning. On the basis of CX3CL1-CX3CR1 signaling, astrocytes trigger WNT signaling to reduce neuronal contact before microglial phagocytosis, thereby creating an optimal environment for microglia-mediated synaptic pruning [207]. Furthermore, astrocyte-secreted cytokines have been shown to modulate microglial states to mediate synaptic pruning. Under physiological neurodevelopmental conditions, IL-33 secreted by astrocytes signals synaptic pruning to microglia [208]. Secreted astrocyte-derived CCN1 acts on SDC4 in microglia to increase lipid storage. Pathologically, reduced astrocyte CCN1 secretion leads to hyperactivated microglia with impaired phagocytic capacity, ultimately preventing the repair of damaged white matter in the brain [209].

In addition to providing evidence supporting the regulation of microglia by astrocytes, microglia have been shown to regulate astrocyte proliferation and development and maintain their normal structure and supportive functions. For instance, during neurodevelopment, the transcriptional repressor Bach1 regulates the expression of the key glycolytic enzymes HK2 and GAPDH, mediating changes in lactate and histone lactylation levels. The level of LRRC15 is regulated by this process and acts on the astrocytic surface receptor CD248 to activate the JAK/STAT pathway, thereby mediating astrocyte proliferation and development [210]. Furthermore, Duo et al. reported that after CNS microglia were cleared using CSF1R antagonists, the reduced expression of astrocytic gap junctions Cx30 and Cx43 severely disrupted functional syncytia, impeding intercellular ion and metabolite exchange. This compromises fundamental synaptic transmission strength and plasticity mechanisms such as long-term potentiation, indicating that the presence of microglia is fundamental for astrocytes to maintain their support of neuronal electrophysiological function [211].

Several studies have identified factors that disrupt this homeostasis. For example, a high-salt diet triggers the localized accumulation of reactive microglia around vasopressin-secreting neurons, which phagocytose the astrocytes covering the neurons in this region. This prevents the clearance of glutamate released by neurons, leading to NMDA receptor activation and neuronal stress [212].

#### Complex interactions between microglia and astrocytes under inflammatory conditions

Beyond immune-mediated synaptic pruning, increasing attention has focused on the synergistic activation and interplay between microglia and astrocytes in response to inflammatory stimuli. In a phenotypic study using microfluidic devices, Zhu et al. reported that when astrocytes in the central compartment were exposed to LPS, microglia migrated toward this compartment, accompanied by proinflammatory polarization and enhanced motility [213]. Numerous studies have confirmed that at the mechanistic level, cytokines, chemokines, and their receptors remain central to this cooperative response, and direct cell-to-cell contact-mediated signaling between these two glial cell types has been identified.

In various neuroinflammatory contexts, the amplification of inflammatory signals through these interaction modes is considered a key pathogenic driver in multiple diseases. For example, CXCL12, which is released by microglia in a paracrine manner or by astrocytes in an autocrine manner, activates astrocytes via CXCR4. Activated astrocytes then release TNF-α, which in turn activates microglia [214]. This synergistic activation exacerbates central immune disorders such as MS [215]. Furthermore, Rothhammer et al. demonstrated that in autoimmune encephalitis, microglia secrete VEGF-B signals through FLT-1 expressed on astrocytes, driving them into a proinflammatory state and thereby aggravating disease progression [216]. In chronic neuropathic pain, which has been historically attributed largely to astrocyte activation, recent evidence has indicated that microglia–astrocyte interactions amplify C-fiber synaptic strength, thereby enhancing hyperalgesia [217]. Signaling between microglia-derived IL-18 and astrocytic IL-18R has been proposed as a potential mechanism underlying this process [218]. In AD, IFN-γ secreted by reactive astrocytes upregulates Keap1 in microglia, reducing NRF2 nuclear translocation and anti-inflammatory gene transcription while simultaneously upregulating NFκB to promote microglial activation and neuroinflammation [219].

Interactions between microglia and astrocytes are not exclusively proinflammatory, whereas certain pathways mediate synergistic resolution of inflammation. Microglia adopting a proresolution phenotype secrete the anti-inflammatory cytokine IL-10, which acts on IL-10R1 in astrocytes, promoting their transition to a proresolution phenotype. These astrocytes then secrete TNF-β, establishing a positive feedback loop that promotes inflammation resolution [220]. In MS, the upregulation of PD-L1 expression on astrocytes and PD-1 expression on activated microglia creates a local glial immune checkpoint that transmits anti-inflammatory signals to microglia [221]. Moreover, in autoimmune encephalomyelitis, activated microglia can exert neuroprotective effects by secreting TGF-α, which acts on the ErbB1 receptor of astrocytes and limits their pathogenic activity [216].

Notably, the relationship between microglia and astrocytes is not only synergistic but also involves complex, context-dependent, and sometimes antagonistic interactions. For instance, in microfluidic coculture systems, compared with microglia alone, LPS-stimulated microglia cultured with astrocytes secreted lower levels of proinflammatory factors but showed increased complement C3 secretion [222]. Such opposing effects may correlate with disease stage or temporal dynamics. During the acute phase of intracerebral hemorrhage, microglia secrete IGF1 and OPN to activate the mTOR pathway in astrocytes, promoting the formation of a protective scar. Over time, this initial protective response becomes detrimental, and delayed microglial clearance in later stages prolongs tissue damage [223]. Cortical spreading depolarization induces significant cellular stress in the CNS, triggering intense inflammation without cell death [224]. Under these conditions, the release of chemokines from neurons initially ceases, followed by the nuclear translocation of NF-κB in nearby astrocytes, which shift from a proinflammatory phenotype to a proresolution phenotype. Concurrently, microglia upregulate genes related to chemotaxis (*CCL3*) and synaptic pruning (*C1Q*), adopting a transcriptional profile consistent with a proresolution phenotype [225].

In addition to classical signaling pathways, exosomes have been implicated in the immunoregulatory network between astrocytes and microglia. For example, microglia activated by pathological protein aggregates induce proinflammatory astrocyte activation via peli1-mediated exosome secretion [226]. During neuroinflammation, astrocytes release ATP/ADP through vesicular export, triggering P2Y12- and VNUT-dependent activation and migration of microglia, which in turn release additional ATP/ADP, perpetuating the response [227].

In summary, astrocyte‒microglia interactions do not follow a simple command–execute hierarchy but constitute a dynamic, bidirectional molecular network modulated by other cell types. This network operates through cytokine signaling, direct contact, and exosomal communication, and its configuration evolves with disease stage, microenvironmental cues, and temporal progression. Notably, the functional outcomes of reactive astrocytes are highly context dependent and cannot be simply categorized as strictly beneficial or detrimental; the phenotypic evolution of both cell types under neuroinflammatory conditions remains incompletely understood. However, with advances in single-cell and spatial transcriptomics, a more refined understanding of this complex system is anticipated.

### Microglia‒oligodendrocyte cross-talk

Oligodendrocytes extend their processes to wrap neuronal axons, forming a multilayered lipid membrane structure known as the myelin sheath. This sheath provides electrical insulation, ensuring efficient propagation of action potentials along axons. In addition to myelination, oligodendrocytes also supply metabolic and trophic support to axons [228]. In various neurological disorders, such as MS and AD, myelin destruction and loss—termed demyelination—constitute a core pathological feature [229]. Demyelination arises from two principal mechanisms: (1) direct toxicity to oligodendrocytes by pathological proteins such as α-synuclein and Aβ [230] and (2) excessive phagocytosis or signaling driven by activated microglia under neuroinflammatory conditions. Notably, in proteinopathy-associated diseases, both mechanisms often coexist, whereas in neuroimmune disorders, the latter may act as an independent determinant. Thus, the interaction between microglia and oligodendrocytes can critically influence myelin integrity.

As with other glial interactions, cytokine signaling and transmembrane protein engagement play central roles in microglia‒oligodendrocyte crosstalk. In AD, downregulation of the microglial-activating transmembrane protein DAP12 is correlated with myelin loss in mice [231]. Similarly, the expression of macrophage scavenger receptor 1 (MSR1) is elevated in microglia from patients with AD, dementia with Lewy bodies (DLB), and PD. MSR1-overexpressing microglia exhibit hyperactivation and enhanced myelin phagocytosis [232]. Aging, a key driver of neurodegenerative progression, is accompanied by chronic microglial activation. Activated microglia in aged brains are physically associated with white matter tracts and are correlated with a reduction in myelin [233]. Mechanistically, aged microglia secrete CXCL10, recruiting CD8⁺ T cells to white matter and synergistically promoting myelin damage [234]. Similarly, CNS infection with HSV-1 triggers antiviral and proinflammatory changes in microglia, which recruit peripheral immune cells to participate in demyelination, a process also mediated by microglial cytokines [235]. In ICH, LCN2-positive microglia secrete CSF1, which acts on CSF1R in oligodendrocytes and induces ferroptosis in these cells [236].

However, conflicting evidence regarding CSF1R signaling warrants attention. Wlodarczyk et al. reported that microglia-derived CSF-1 and IL-34, both of which act through oligodendrocytic CSF1R, reduce demyelination and alleviate autoimmune encephalomyelitis [237]. Similar contradictions exist for TGF-β signaling. One study revealed that microglial TGF-β maintains a controlled response state via autocrine mechanisms in response to age-related demyelination stimuli and counteracts myelin degeneration in aging models [238]. In a mouse model of MS, however, Western-style diet-enhanced TGF-β signaling suppressed LXR activity, inhibiting myelin phagocytosis and cholesterol clearance. TGF-β inhibition restored the ability of microglia to clear myelin debris [239]. Conversely, TGF-β receptor inhibition reduced demyelination in a zebrafish model of MS [240]. Moreover, microglial TGF-β signaling drives oligodendrocyte dysfunction in individuals with alcohol use disorder [241]. These discrepancies highlight the need to clarify the context-dependent roles of CSF-1 and TGF-β in regulating microglial myelin phagocytosis. First, future studies should prioritize disease-specific conditions. For instance, in acute injury, strongly proinflammatory microglia may mediate CSF1 and TGF-β signaling, which exacerbates demyelination, whereas in chronic neuroinflammation, specific microglial subsets may promote myelin protection via the same pathways. Second, dose‒response relationships between cytokine concentrations and receptor activation must be delineated, as different signaling intensities may produce opposing outcomes. Finally, the differential effects of receptor subtypes deserve particular attention; e.g., the TNF receptors TNFR1 and TNFR2 have opposing effects on oligodendrocyte survival and function [242].

### Microglia–T-cell crosstalk

#### T-cell recruitment signals

**Fig. 3:**
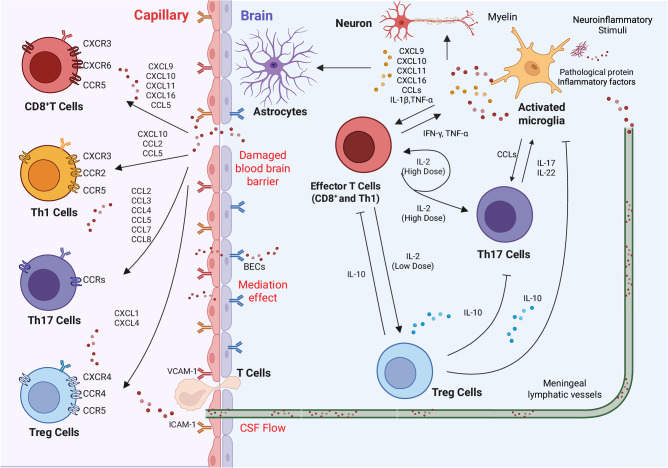
Orchestration of T-cell trafficking and neuroinflammatory crosstalk across the blood‒brain barrier (BBB). This schematic illustrates the dynamic recruitment of T-cell subsets and their subsequent effector functions within the CNS microenvironment. Peripheral activation and recruitment: In the capillary compartment, various T-cell subsets—including CD8⁺ T cells, Th1 cells, Th17 cells, and Tregs—express specific chemokine receptors (e.g., CXCR3, CXCR4, CCR2, and CCR5). These cells are recruited to the CNS in response to gradients of chemokines (such as CXCL9/10/11 and CCL2/5) secreted by activated brain endothelial cells and astrocytes. Transmigration across the damaged BBB: Neuroinflammatory stimuli and pathological factors trigger the upregulation of adhesion molecules (VCAM-1 and ICAM-1) on brain endothelial cells, facilitating T-cell adhesion and diapedesis across a compromised BBB. Intraparenchymal neuroinflammation: Upon entry, effector T cells (CD8⁺ and Th1) and Th17 cells interact with activated microglia and astrocytes. Proinflammatory cytokines (e.g., IFN-γ, TNF-α, IL-17, and IL-22) and inflammatory factors further exacerbate microglial activation and contribute to neuronal damage and myelin degradation. IL-2–Treg regulatory axis: The local cytokine milieu significantly modulates immune homeostasis. While high-dose IL-2 promotes the expansion of effector T cells and Th17 cells, low-dose IL-2 selectively supports the proliferation and stability of Tregs. These Tregs exert neuroprotective effects through the secretion of anti-inflammatory cytokines such as IL-10, which antagonize the proinflammatory activities of Th17 and effector T cells. CSF and lymphatic clearance: The diagram also depicts the circulation of T cells within the CSF flow and their eventual exit through the meningeal lymphatic vessels, highlighting the integrated communication between the CNS and the peripheral lymphatic system. The figure was created with BioRender.com

T cells are the core cellular components of adaptive immunity and are broadly classified into CD4⁺ and CD8⁺ subsets on the basis of surface markers and functional profiles [243]. CD4⁺ T cells perform helper, proinflammatory, and regulatory functions through cytokine secretion, with specific roles determined by differentiation cues—for example, IL‑12 and IFN‑γ drive Th1 differentiation for cellular immunity, whereas IL‑4 promotes Th2 differentiation for humoral responses [244]. CD8⁺ T cells recognize target cells and exert cytotoxicity via perforin/granzyme release and the Fas/FasL pathway [245]. Additional subsets include memory T cells and unconventional populations such as γδ T cells and NK-T cells [246, 247]. Although the CNS has long been considered immune‑privileged, emerging evidence implicates T-cell activation and dysfunction in various neuroinflammatory diseases, highlighting the importance of CNS–peripheral immune crosstalk. Recent studies have delineated pathways through which T cells participate in neuroinflammation. Resting microglia that perform immune surveillance become activated upon stimulation (e.g., by IFN‑γ), upregulating the expression of MHC class I/II and costimulatory molecules [248, 249]. After they phagocytose and process antigens, they present antigenic peptides on MHC molecules and secrete inflammatory cytokines and chemokines that recruit activated T cells across the BBB [250, 251]. Owing to ligand–receptor specificity, distinct chemokines recruit different T‑cell subsets (Fig. 3).

The CXCL10-CXCR3 axis is key for CD8⁺ T‑cell recruitment. In experimental cerebral malaria, CXCL10^high^TNF-α^high^ microglia activate and recruit CD8⁺ T cells [252]. Aging‑related studies have further shown that chronically activated microglia recruit CD8⁺ T cells via this axis to attack myelin [234]. More direct evidence comes from a 3-D neuroimmune model using cells derived from AD patients, in which microglia-secreted CXCL10 induced the migration of CXCR3⁺ CD8⁺ T cells (but not B cells) into the CNS, triggering further microglial activation and exacerbating neuroinflammation [253]. Additional CXCL-CXCR axes involved in CD8⁺ T‑cell activation and CNS recruitment include CXCL9/CXCL11 (both of which act on CXCR3) and CXCL16 (which acts on CXCR6) [254–257]. Notably, CCL5, another potent CD8⁺ T‑cell chemoattractant, is also secreted by activated microglia [258]. Cerebrospinal fluid (CSF) levels of CCL5, CXCL9, and CXCL10 are elevated in MS patients [259], and pharmacological blockade of CCL5 attenuates Aβ deposition in AD models [260]. However, more direct functional evidence (e.g., conditional genetic manipulation) is needed to establish a causal role for CCL5 in mediating peripheral immune cell infiltration. Other chemokines, such as CCL3 and CCL4, which act synergistically with CCL5, also warrant further investigation.

In addition to CD8⁺ T cells, specific CD4⁺ subsets are recruited to the CNS, where they either exacerbate or counteract neuroinflammation. Th1 cell recruitment is mediated by CXCL10-CXCR3 [261], CCL2-CCR2 [262], and CCL5-CCR5 [263, 264]. Once recruited, Th1 cells amplify inflammation by secreting IFN‑γ, which further activates and sustains microglial function [265, 266]. Th17 cells, which secrete IL‑17 A, IL‑17 F, and IL‑22, migrate to the CNS and contribute to demyelination. Chemotactic factors for Th17 migration include CCL2, CCL3, CCL4, CCL7, and CCL8 [267], with Ccl5 also being involved [268]. After they enter the CNS, Th17 cells engage in synergistic inflammatory feedback with microglia via MHC‑II expression, proinflammatory cytokine/chemokine secretion, and STING/NF‑κB pathway activation [269]. T follicular helper (Tfh) cells, another effector CD4⁺ subset, have also been shown to infiltrate the CNS in glia‑associated contexts [270, 271]. ICOS/ICOS-L signaling supports Tfh survival, expansion, or function within the CNS [272], and correlation analyses suggest a positive association between CCR5 expression and Tfh immune infiltration [273].

In temporal lobe epilepsy, an imbalance in the peripheral Th1/Th2 ratio is observed, with elevated levels of Th1 cytokines (IFN‑γ and TNF‑α) and reduced levels of Th2 cytokines (IL‑4 and IL‑10) [274]. As functional antagonists of Th1 cells, Th2 responses are suppressed during inflammatory surges. Restoring Th2 function or enhancing Th2 recruitment to counter Th1‑mediated neuroinflammation represents a potential therapeutic strategy. In spinal cord‑injured mice, the administration of IL‑10‑expressing mesenchymal stem cells increased the numbers of proresolution-phenotype microglia, regulatory T cells (Tregs), and Th2 cells at the injury site while reducing the number of Th1 cells and promoting neuroregeneration [275]. However, blockade of Th2 cytokines (IL‑4, IL‑5, IL‑9, and IL‑13) in ischemic brain injury did not affect histopathology [276]. Although the latter study lacked a positive control for Th2 stimulation, the conflicting results underscore the need for more direct investigations of Th2 involvement in neuroinflammation.

In contrast to Th2 cells, Tregs are well‑established anti‑inflammatory CD4⁺ subsets. Treg infiltration and anti‑inflammatory factor secretion have been documented in patients with spinal cord injury and ischemic stroke. Treg-secreted osteopontin inhibits CD74 expression in microglia and activates integrin receptors to increase the phagocytosis of damaged synapses [277, 278]. Tregs also mitigate microglial inflammation via CTLA‑4 and upregulate ABCG1 in microglia to manage myelin load and reduce lipid droplet formation [279]. In traumatic brain injury, intranasal anti‑CD3 antibody treatment induces the migration of IL‑10‑producing Tregs to the brain, where they interact with microglia and limit neuroinflammation; moreover, IL‑10 depletion abrogates these benefits [280]. In ischemic stroke, the CXCR4-CXCL1 axis is a potential pathway for Treg CNS migration [281, 282]. Enhanced CNS migration is observed in CCR4‑overexpressing Tregs, suggesting that CCL4-CCR4 is another recruitment route [283]. Nevertheless, systematic studies identifying the core chemokines involved in the recruitment of Tregs to the CNS remain limited.

Notably, low-dose IL-2 therapy has recently shown promise in modulating Tregs to control neuroinflammatory diseases. A high-calorie diet reduces the number of hypothalamic Tregs, whereas low-dose IL-2 supplements them, limiting hypothalamic immune activation and reversing metabolic dysfunction [284]. In autistic mice, low-dose IL-2 reprograms microglia toward a pro-resolution phenotype and alleviates core symptoms [270]. A small clinical trial in AD patients reported reduced CNS inflammatory markers and Aβ levels after low‑dose IL‑2 treatment [285]. Two important considerations are that (1) high-dose IL-2 activates effector T cells such as CD8⁺ T cells [286], and (2) IL-2 is secreted by activated CD4⁺ T cells but not microglia [287]. On the basis of the current evidence, we propose the following integrated pathway: under neuroinflammation, activated microglia secrete chemokines (e.g., CXCL/CCR family members) that specifically recruit proinflammatory T cells (CD8⁺, Th1, and Th17 cells) into the CNS, initiating a migratory cascade [234]. Recruited, activated CD4⁺ T cells secrete IL‑2, which is preferentially utilized by effector T cells during acute inflammation, driving a proinflammatory positive feedback loop [288, 289]. As the disease progresses to recovery, the microenvironment shifts IL‑2 signaling toward regulatory T (Treg) homeostasis and function [290, 291]. Tregs proliferate, migrate to the CNS, and secrete anti‑inflammatory factors such as IL‑10, promoting microglial polarization toward an anti‑inflammatory phenotype. A comprehensive experiment is needed to verify this hypothesis regarding this circuit. Defining IL‑2 concentration thresholds that selectively activate Tregs and subsequently tailor IL‑2 dosing accordingly represents a promising direction for clinical translation.

#### T‑cell recruitment pathways

Chemokines secreted by activated microglia must physically interact with T cells to facilitate their migration. However, most chemokine proteins cannot freely diffuse across an intact, healthy BBB. Although BBB disruption in acute or chronic neuroinflammatory conditions—such as hemorrhagic stroke or aging—may allow some chemokine leakage [292, 293], this process depends on severe cerebrovascular endothelial damage and is not the dominant mode of signaling. Recent studies have clarified the mediating roles of cerebrovascular endothelial cells and astrocytes. Microglial chemokines and inflammatory factors can activate endothelial cells and astrocytes, leading to the upregulation of the expression of adhesion molecules such as VCAM‑1 and ICAM‑1 and the luminal display of specific chemokines that recruit distinct T‑cell subsets [294, 295].

Nevertheless, this mechanism alone cannot explain the elevated levels of multiple chemokines—including CCL2, CCL5, CXCL9, CXCL10, CXCL13, IL‑16, and CXCL1—observed in the CSF of MS patients [296, 297]. In 2015, Louveau et al. identified functional lymphatic vessels lining the dural sinuses, providing an anatomical pathway through which microglia-derived chemokines bypass the BBB [298]. Subsequently, Xu et al. demonstrated that inflammatory mediators and chemokines can drain via these lymphatic vessels through the CSF to deep cervical lymph nodes, where they directly contact and activate naïve T cells, driving their differentiation into effector cells with CNS-homing capacity [299]. This delineation of a central-lymphatic-immune circuit explains the persistent increase in CSF chemokine profiles in neuroinflammatory diseases and provides a theoretical foundation for further exploration of CNS-peripheral immune crosstalk and therapeutic strategies targeting the lymphatic-immune interface.

In summary, microglia–T-cell interactions represent a precise yet potentially hazardous double‑edged sword. Under homeostatic conditions, microglia contribute to essential immune surveillance through basal antigen presentation and limited chemokine secretion. At this stage, a small number of infiltrating T cells—particularly regulatory T cells—help clear abnormal proteins, promote tissue repair, and exert neuroprotective effects. Under pathological stimulation, however, this interaction rapidly transforms into a potent inflammatory amplifier. Activated microglia precisely recruit proinflammatory T‑cell subsets—including CD8⁺ cytotoxic T cells and Th1 and Th17 cells—across the BBB via specific chemokine axes (e.g., CXCL10/CXCR3 and CCL5/CCR5) and CSF-lymphatic drainage pathways. These infiltrating T cells further exacerbate the inflammatory phenotype of microglia through the secretion of cytokines such as IFN‑γ and IL‑17, prompting the release of additional chemokines and the production of neurotoxic mediators. This establishes a self‑perpetuating positive feedback loop that drives neuronal injury, synaptic loss, and myelin destruction—a process that is particularly prominent in chronic neurodegenerative and inflammatory diseases such as AD and MS. Therefore, elucidating the specific molecular pathways and spatiotemporal dynamics of microglia‒T-cell crosstalk and developing strategies that precisely disrupt their harmful circuits while preserving or enhancing their protective functions represent some of the most promising directions in neuroimmunotherapy.

### Emerging roles of B cells in microglia

Although the mechanisms underlying B‑cell involvement in CNS inflammation are less well characterized than those underlying T-cell involvement, emerging evidence supports their role in several neuroinflammatory conditions. For instance, a genome‑wide and brain‑wide genetic and transcriptomic analysis revealed a significant association between B-cell signatures and MS [300]. In an aging mouse model with circadian disruption, increased splenic B-cell numbers correlated negatively with cognitive decline [301]. In AD, memory B cells accumulate in the lung, and antagonizing their function with anti‑CD40 antibodies exacerbates pathology, whereas enhanced pulmonary B‑cell activation alleviates disease manifestations. Subsequent work has shown that microglial-derived CXCL12 recruits memory B cells to the CNS via CXCR4 [302]. Furthermore, Touil et al. reported that in the meninges of MS patients, proinflammatory effector B cells induce microglia to secrete inflammatory mediators (e.g., IL‑12, IL‑6, and TNF-α) while downregulating the expression of anti‑inflammatory factors such as IL‑10 [303]. Collectively, these findings point to a modulatory role for B cells in neuroinflammation, although the specific contexts, recruitment pathways, and effector mechanisms involved remain to be fully elucidated.

## Summary and outlook

This review systematically elucidates the central role and dynamic complexity of microglia, as resident immune sentinels of the CNS, in neuroinflammation. From developmental origins to lifelong maintenance and from the classical binary activation paradigm to the rich spectrum of states (such as DAM, ARM, and IRM) revealed by single‑cell technologies, microglia exhibit a functional diversity that far exceeds the simplistic dichotomy of “resting” versus “activated” states. In various neuroinflammatory diseases, microglial responses share common activation features—such as the cascade release of proinflammatory mediators, complement‑mediated excessive synaptic pruning, and activation of pyroptosis pathways—while also displaying significant disease‑specific heterogeneity. In neurodegenerative diseases, this heterogeneity is primarily reflected in the convergence of numerous disease‑risk genes in microglial phagocytic and lysosomal pathways, profoundly influencing their ability to clear pathological proteins and thereby modulating disease progression. More importantly, it is essential to emphasize that microglia do not act in isolation. Through cytokines, chemokines, direct contact, and exosomes, they form an intricate and dynamic interactive network with neurons, astrocytes, oligodendrocytes, recruited T cells, and potentially B cells. Under homeostasis, this network maintains synaptic plasticity, myelin integrity, and tissue repair, whereas in pathological states, it transforms into a positive feedback loop that drives disease progression.

Looking ahead, research on microglia is transitioning from describing phenomena to precise intervention. First, moving beyond the simple “pro‑inflammatory/anti‑inflammatory” dichotomy toward a deeper understanding of how specific microenvironmental signals drive microglia toward distinct functional states (e.g., neuroprotective DAMs or phagocytically specialized subsets) will be key to developing targeted therapies. Second, the use of human iPSC‑derived organoids and high‑resolution spatial multi‑omics technologies will enable the simulation and analysis of the spatiotemporal dynamics of microglial interactions with other cells in environments more closely resembling the human brain, thereby revealing novel regulatory nodes. Furthermore, given the precise chemokine‑receptor dialogs (e.g., the CXCL10‑CXCR3 axis) and CSF‑lymphatic drainage pathways between microglia and peripheral immune cells such as T cells, intervening in these specific recruitment pathways—rather than employing global immunosuppression—is promising for controlling neuroinflammation while minimizing systemic side effects. In summary, leveraging the characteristics of microglia—their high heterogeneity, plasticity, and position as central hubs within complex communication networks—and employing spatiotemporally precise regulatory strategies to steer their functions toward beneficial outcomes and away from detrimental outcomes represents a promising approach for breaking the vicious cycle of neuroinflammation and preserving brain health.
